# Ancient founder mutation is responsible for Imerslund-Gräsbeck Syndrome among diverse ethnicities

**DOI:** 10.1186/1750-1172-6-74

**Published:** 2011-11-13

**Authors:** Cameron M Beech, Sandya Liyanarachchi, Nidhi P Shah, Amy C Sturm, May F Sadiq, Albert de la Chapelle, Stephan M Tanner

**Affiliations:** 1 Comprehensive Cancer Center, The Ohio State University, Columbus, OH 43210, USA; 2 Department of Internal Medicine, The Ohio State University, Columbus, OH 43210, USA; 3 Department of Biological Sciences, Yarmouk University, Irbid 21163, Jordan

**Keywords:** Imerslund-Gräsbeck syndrome, juvenile cobalamin deficiency, founder mutation, age estimation, mutation screening, anemia, ethnicity

## Abstract

**Background:**

Imerslund-Gräsbeck syndrome (IGS) was described just over 50 years ago by Olga Imerslund and Ralph Gräsbeck and colleagues. IGS is caused by specific malabsorption of cobalamin (Cbl) due to bi-allelic mutations in either the cubilin gene (*CUBN*) or the human amnionless homolog (*AMN*). Mutations in the two genes are commonly seen in founder populations or in societies with a high degree of consanguineous marriages. One particular mutation in *AMN*, c.208-2A>G, causing an out-of-frame loss of exon 4 in the mRNA, is responsible for some 15% of IGS cases globally. We present evidence that this founder mutation causes a substantial percentage of cases among diverse ethnicities and that the mutation is as old as human civilization.

**Methods:**

Partial genotyping indicated a founder event but its presence in diverse peoples of Arabic, Turkish, Jewish, and Hispanic ancestry suggested that the mutation might be recurrent. We therefore studied the flanking sequence spanning 3.5 Mb to elucidate the origin of the haplotype and estimate the age of the mutation using a Bayesian inference method based on observed linkage disequilibrium.

**Results:**

The mutation's distribution, the size of the shared haplotype, and estimates of growth rate and carrier frequency indicated that the mutation was a single prehistoric event. Dating back to the ancient Middle East around 11,600 BC, the mutation predates the advent of writing, farming, and the monotheistic religions of the region.

**Conclusions:**

This mutation causes over 50% of the IGS cases among Arabic, Turkish, and Sephardic Jewish families, making it a primary target for genetic screening among diverse IGS cases originating from the Middle East. Thus, rare founder mutations may cause a substantial number of cases, even among diverse ethnicities not usually thought to be related.

## Background

Imerslund-Gräsbeck syndrome (IGS, megaloblastic anemia 1; OMIM261100) is a recessively inherited childhood disorder[1]. IGS is prevalent in societies where consanguinity is common or in communities that underwent a population bottleneck [2]. The disease is manifested by an increased propensity for infections, fatigue, attention deficit, paralysis, and ultimately megaloblastic anemia that can be fatal if left untreated. The primary diagnostic criteria are reduced vitamin B_12 _levels in the serum, elevated homocysteine and methylmalonic acid levels in the blood and urine, and often mild proteinuria [3]. The disorder, which is caused by selective malabsorption of cobalamin (Cbl; vitamin B_12_) in the intestine, occurs due to bi-allelic mutations in either the cubilin (*CUBN*, OMIM602997 [4]) or the amnionless gene (*AMN*, OMIM605799 [2,5]. The proteins encoded by *CUBN *and *AMN *form a cellular receptor named cubam found on the enterocytes in the ileum, renal tubular cells, and cells of the yolk sac. In the intestine, cubam facilitates the absorption of Cbl and other nutrients from the food, and in the kidneys, cubam is responsible for protein reabsorption [6]. Cubam is further required for patterning of the rodent embryo [7] but its role in human development is presently unclear [8]. Interestingly, mutations in the *GIF *gene coding for gastric intrinsic factor have been implicated in Intrinsic Factor Deficiency (IFD; OMIM261000 [9-11]), a disease with similar symptoms except for proteinuria, which is not seen in IFD. Both IGS and IFD are treatable with life-long parenteral Cbl supplementation, which alleviates the symptoms except the treatment-resistant proteinuria in IGS [3].

Diagnosing IGS is a time-consuming and often inconclusive procedure mainly based on excluding other causes of Cbl deficiency, of which there are many [3]. Genetic diagnostics is not widely available and is far from straightforward because of the genetic heterogeneity. We have screened over 150 patients or sibships with recessive hereditary Cbl malabsorption in the past 10 years ([2,5,10,11] and SMT unpublished data). About 80% were mutated in either *AMN *or *CUBN *(causing IGS) or *GIF *(causing IFD), while the *ABCC1 *gene was excluded [12]. Early on, we documented four IGS patients from three families (two Turkish and one Sephardic Jewish) with the homozygous splice site mutation c.208-2A>G in intron 3 of *AMN *[2]. This mutation causes exon 4 (88 bp) to be skipped in the messenger RNA and results in a frameshift that leads to a truncated *AMN *protein [5]. Limited genotyping suggested that these four patients shared a similar haplotype flanking the mutation [2]. Since then we have identified an additional 16 patients from 13 unrelated families, for a total of 20 patients from 16 sibships, all homozygous for the *AMN *c.208-2A>G mutation (Additional file 1). Most families were of Sephardic Jewish (4) or Turkish descent (7) but also included Arabic families from Jordan (3). One case from the USA had Hispanic roots but based on the name was judged to be of Jewish ancestry and one case was from Spain without detailed ethnic information. The occurrence of this particular mutation in patients originating mainly from the Eastern Mediterranean supported that c.208-2A>G might be a founder mutation. However, its incidence among these different ethnicities and across a large geographic region challenged that notion. The alternative explanation was that c.208-2A>G was a recurrent molecular defect, arising repeatedly *de novo*. Given that this mutation is the most frequent cause of IGS outside Scandinavia, clarifying its origin was of clinical importance.

## Methods

### Patient samples and controls

**Figure 1:**
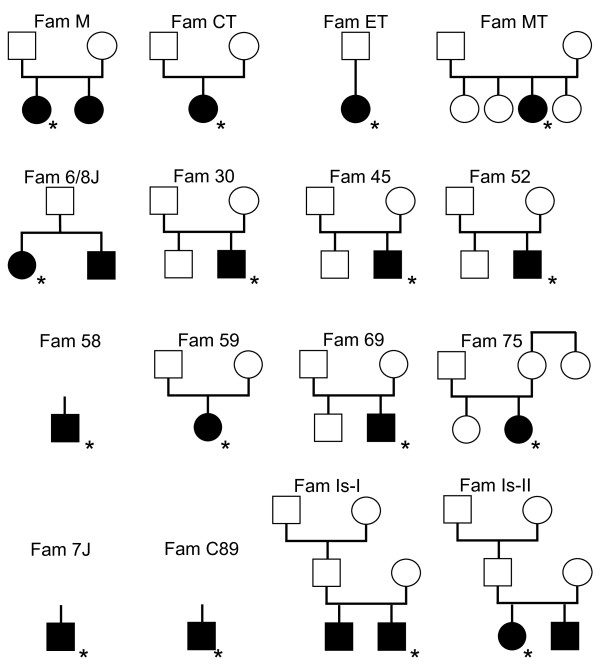
**Pedigrees of 16 sibships with Imerslund-Gräsbeck mutation *AMN *c.208-2A>G**. Full symbols denote patients with IGS. Only individuals from whom DNA was available for mutation analysis and genotyping are shown. The individual from whom a sample was used for the estimation of the age of the mutation is marked by an asterisk.

A total of 20 patients with IGS (9 female, 11 male, ages 2-19 years when diagnosed), 24 parents, 8 unaffected siblings, and 4 grandparents from 16 IGS families were included in this study (Additional file 1 and Figure 1). Patients with IGS were diagnosed based on established criteria [1,13], while the parents were healthy. All the families were allegedly unrelated. Thirty-six anonymous Jordanian controls (Arabs, ages > 18 years) were obtained from the Department of Biological Sciences, Yarmouk University in Irbid, Jordan.

### Mutation screening and genotyping

Genomic DNA was extracted from white blood cells using standard phenol-chloroform-ethanol precipitation after written informed consent according to institutional review board guidelines of the Ohio State University and the Declaration of Helsinki. Molecular diagnosis involved screening the *AMN *and *CUBN *genes for suspected IGS. Individual exons of *AMN *or *CUBN *were amplified by PCR and analyzed as previously described [2,5]. After identifying the *AMN *c.208-2A>G mutation in exon 4, DNA samples from cases and family members were genotyped.

**Figure 2:**
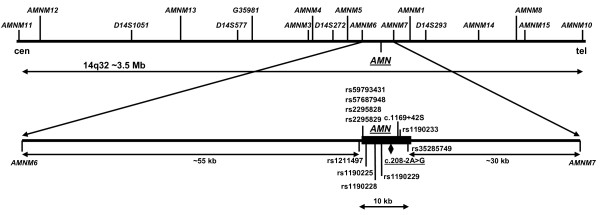
**Map of the *AMN *gene locus in chromosome band 14q32**. Eighteen microsatellite markers flanking *AMN *from 2.3 Mb centromeric to 1.2 Mb telomeric are shown above the upper line. Eleven single nucleotide polymorphisms between *AMNM6 *and *AMNM7 *and centering on the *AMN *gene locus are shown in the enlarged region below. The *AMN *gene is depicted as a black rectangle and the mutation is underlined. All 29 marker loci were used for genotyping and age calculations (see text). Physical distances are not drawn to scale.

Flanking markers from 2.3 Mb centromeric to 1.2 Mb telomeric of the *AMN *locus were genotyped, including 18 microsatellites (*AMNM11*, *AMNM12*, *D14S1051*, *AMNM13*, *D14S577*, *G35981*, *AMNM3*, *AMNM4*, *D14S272*, *AMNM5*, *AMNM6*, *AMNM7*, *AMNM1*, *D14S293*, *AMNM14*, *AMNM8*, *AMNM15*, *AMNM10*) and 11 single nucleotide polymorphisms (rs1211497, rs59793431, rs57687948, rs2295828, rs2295829, rs1190225, rs1190228, rs1190229, c.1169+42S, rs1190233, rs35285749; Figure 2). The microsatellites labeled *AMNMn *and *G35981 *were simple-sequence tandem-repeats identified by repeat masker. The microsatellites were genotyped using FAM-labeled primers and the Genotyper program, while the SNPs were typed by SNaPshot (Applied Biosystems, Foster City, CA).

### Haplotype construction

Obtained genotype data were used to construct haplotypes using the linkage program Merlin [14] and the PHASE 2.0 program [15] according to their manuals.

### Age estimation

Mutation age estimate was carried out with the DMLE+2.3 software program [16] using the 29 loci genotyped. In order to do so, the proportion of disease haplotypes sampled and the growth rate were estimated. For the Sephardim, we used an estimate of ~2 million for its population, while for the Muslim source population we added up Turkey (~76.5 million) and Jordan (~6.5 million), for a total of 83 million [17]. The proportion of disease chromosomes sampled depended on an estimate of the mutation frequency, which was unknown. Thus, the incidence rate of IGS was estimated at 1 case per 10,000, 1:100,000, 1:500,000 and 1:1,000,000 people for a calculated carrier frequency of the mutation of 1 in 100, 1 in 316, 1 in 707, and 1 in 1,000 individuals, respectively. Turkey has a current population growth rate of 1.24% and Jordan's is 0.98% per year [17]. Before 1 AD the annual population growth rate was potentially less than ~1% [18,19]. Thus, for the growth rate a range of values (1-3%) was used for the calculations in part based on the growth rates by Zelinger and colleagues [20].

## Results & Discussion

### Mutation haplotype

The haplotyping results indicated that two almost identical haplotypes carried the mutation. One was found in the 5 "Jewish" families (4 Sephardic and one Hispanic) and the other in the 10 "Muslim" (Turkish and Jordanian) families. The sole Spanish case (patient family 30; Figure 1) carried a "Jewish" and a "Muslim" haplotype. The Jewish haplotype differed from the Muslim haplotype - with a few exceptions - at marker *D14S1051 *(genotype 182/180), two SNPs upstream of exon 1 (rs2295828, rs2295829), and marker *AMNM1 *(192/190); i.e. the Jewish haplotype is 182-C-C-192 and the Muslim haplotype is 180-T-G-190 (Additional file 1 and Figure 2). At first, this suggested that we observed separate Jewish and Muslim mutational events, in particular because the two SNPs are only 5.8 kb upstream from the mutation c.208-2A>G. However, the two haplotypes shared all alleles up to marker *D14S272*, which is 117 kb centromeric to the mutation, and likewise 60 kb telomeric to the mutation marker *D14S293 *delimited a common conserved haplotype. Moreover, alleles from *AMNM13 *to *AMNM14 *were by and large identical on both haplotypes, indicating that a mutation proto-haplotype of at least 950 kb was shared (Additional file 1 and Figure 2). This fact supported the concept that we observed a single mutational event and that the microsatellite markers *D14S1051*, *AMNM1*, and the two SNPs (rs2295828, rs2295829) were mutated or recombined later.

### Age of the mutation

We first combined the two ethnic groups for age estimation. Of the 20 patients, 16 (5 Jewish, 10 Muslim, and one compound Spanish case) were unrelated and selected for age calculation, resulting in 32 disease chromosomes. The sum of the populations was 85 million. We also concluded that the frequency of IGS is likely greater than 1 in 1 million; at least among the Sephardic Jews with 5 confirmed patients among ~2 million people. The 10 cases among 83 million Turks and Jordanians are probably underestimated because of lack of local genetic testing. We noted that four of the ten Muslim cases were diagnosed among expatriates in Europe. Thus, we estimate the IGS incidence for both groups caused by this mutation to be around 1 in 500,000 people for a carrier frequency of 1:707 but we calculated the age for various incidences (Additional file 2). After applying the different growth rates, the age was estimated to be between 8,050 years (95% confidence interval [CI] 6,450-11,200 years) and 19,225 years (95% CI: 14,300-28,600 years) for an average of ~13,600 years (~11,600 BC).

If the mutation originated around 11,600 BC it could explain why it is today seen in the peoples of Turkey and Jordan but not necessarily why it is only present in Jews of Sephardic roots. We would expect the mutation to be present in various Jewish groups because of their common roots [21,22]. However, to date we have only studied three other unrelated Jewish IGS patients, who were all Ashkenazi. Two of these carried a homozygous *CUBN *mutation, c.2614_2615delGA; p.D872fs (SMT unpublished data), and one was compound heterozygous for *AMN *c.43+1G>T; splice site & c.701G>T; C234F [23]. Since Ashkenazim are generally aware of modern genetic research, the absence of the *AMN *mutation c.208-2A>G among them suggested that it was never present in their founder population. The lack of c.208-2A>G in other Jewish groups of the Diaspora could have a similar explanation. Consequently, the mutation's exclusive presence among Sephardic Jews suggested that the mutation had entered that Jewish group from a non-Jewish background rather than the other way. When the age calculations were repeated by separating the cohort into Jews (2 million and 10 chromosomes) and Muslims (83 million and 20 chromosomes) - the mixed case was removed - we estimated the age to be between 4,275 years (95% CI: 2,875-7,475 years) and 10,975 years (95% CI: 5,525-19,975 years) for the Jewish haplotype and between 7,225 years (95% CI: 5,600-9,875 years) and 19,000 years (95% CI: 13,625-26,650 years) for the Muslim haplotype (Additional file 2). Thus indeed, the Muslim haplotype appeared to be older than the Jewish haplotype. However, the Jewish population was much smaller, thus the proportion of disease chromosomes sampled became greater. This caused the Jewish haplotype to appear younger than it probably is (Additional file 2). The size of the conserved haplotype (when ignoring *D14S1051*, *AMNM1*, and the two SNPs rs2295828 and rs2295829) was about the same in both groups (~1 Mb), suggesting a similar age and origin. The alternative explanation of two identical mutational events in Jews and Muslims would have to assume that the mutation occurred twice around the same time, judged from the haplotype size. Moreover, it would have to have affected two almost identical haplotypes. When studying the frequency of the proto-haplotype among 36 Jordanian controls, we detected it only once among 72 chromosomes (Additional file 1). Thus, the probability of two identical mutations occurring around the same time on the same rather rare haplotype is low. In addition, this mutational event has apparently never happened again, thus the evidence was not supporting a mutational hotspot mechanism. In fact, the consistent differences between the Muslim and the Sephardic haplotypes at *D14S1051*, *AMNM1*, and the two SNPs rs2295828 and rs2295829 point to single split and no subsequent convergence until today. The "compound heterozygous" Spanish patient (family 30) indeed may be the product of Sephardic and later Muslim migration to Spain [24]. The Hispanic case from the USA might also be traced back to the Iberian Peninsula and the expulsion of Jews from there in 1492 AD, which also brought them to the New World. Finally, two more IGS cases with this mutation were described in the literature, one from Tunisia, albeit without further ethnic details [25], and another from Austria with Turkish ancestry [26].

The oldest known founder mutation to date appears to be the deltaF508 in the cystic fibrosis gene *CFTR *that was estimated to be 11,000-52,000 years old depending on genetic (microsatellite mutation rate and selection) and demographic (growth rate and population size) parameters [27,28] but its population origin remains enigmatic [29]. While certain *CFTR *mutations are hypothesized to convey a selective advantage [30], to our knowledge there is no known benefit for healthy heterozygous carriers of IGS mutations, thus a heterosis effect is unlikely. While the exact frequency of IGS carriers remains unknown, the incidence of IGS is probably higher than one would expect from the carrier frequency because of a high degree of consanguinity.

## Conclusions

We have used a series of assumptions to estimate the age range of a founder mutation in *AMN *that causes IGS. Judging the data conservatively, we believe that the region of origin for the mutation to be in Northern Mesopotamia (today's Eastern Turkey and Northern Iraq) some 13,600 years ago, placing it before the beginning of the Neolithic period (ca. 9,500 BC). Its exclusive presence in Sephardic Jews today could be explained by a common ancestor in an early Semitic population whose descendants contributed to that Jewish tribe as well as the Turkish and Arabic populations we recognize today. The natural history of this mutation is in agreement with recent studies that support a common but complex ancestry between Jews and non-Jews in the Middle East [21,22].

Although *AMN *c.208-2A>G is apparently younger than *CFTR *deltaF508, it appears to be one of the oldest human disease mutations known to date and clearly occurred first in the Middle East. It causes some 15% of IGS cases worldwide and more than 50% among Turks, Jordanians, and Sephardim combined (SMT unpublished data). Given that the genetic analysis for newly diagnosed IGS cases is complex, *AMN *c.208-2A>G should be considered first when dealing with patients originating from Turkey, Jordan, Spain, Tunisia, or with an ethnic Sephardic background.

## Competing interests

The authors declare that they have no competing interests.

## Authors' contributions

CMB carried out the molecular genetic studies and drafted the manuscript. SL designed and performed the statistical analysis and helped to draft the manuscript. NPS participated in the molecular genetic studies. ACS and MFS coordinated DNA sample collection. AdlC commented on the manuscript draft. SMT conceived and designed the study, coordinated the research, and wrote the final manuscript. All authors read and approved the final manuscript.

## Supplementary Material

Additional file 1 **Genotypes of 16 sibships with *AMN *mutation c.208-2A>G and haplotype information of 36 Jordanian controls**. Detailed genotype and haplotype data of 29 markers flanking the *AMN *gene. The mutation haplotypes for Jewish and Muslim patients are identified by color. Click here for file

Additional file 2 **Age estimation with DMLE+2.3 for *AMN *mutation c.208-2A>G**. Age estimation calculations based on various growth rates and carrier frequencies as indicated in the text. Click here for file
